# X Chromosome Crossover Formation and Genome Stability in *Caenorhabditis elegans* Are Independently Regulated by *xnd-1*

**DOI:** 10.1534/g3.116.035725

**Published:** 2016-09-27

**Authors:** T. Brooke McClendon, Rana Mainpal, Francis R. G. Amrit, Michael W. Krause, Arjumand Ghazi, Judith L. Yanowitz

**Affiliations:** * Molecular Genetics and Developmental Biology Graduate Program, University of Pittsburgh School of Medicine, Pennsylvania; ‡ Magee-Womens Research Institute, Department of Obstetrics, Gynecology, and Reproductive Services University of Pittsburgh School of Medicine, Pennsylvania 15213; † Department of Pediatrics, University of Pittsburgh School of Medicine, Pennsylvania 15224; § Laboratory of Molecular Biology, National Institute of Diabetes and Digestive and Kidney Diseases, Bethesda, Maryland 20892

**Keywords:** meiosis, ATM, Tip60, ionizing radiation, genome instability

## Abstract

The germ line efficiently combats numerous genotoxic insults to ensure the high fidelity propagation of unaltered genomic information across generations. Yet, germ cells in most metazoans also intentionally create double-strand breaks (DSBs) to promote DNA exchange between parental chromosomes, a process known as crossing over. Homologous recombination is employed in the repair of both genotoxic lesions and programmed DSBs, and many of the core DNA repair proteins function in both processes. In addition, DNA repair efficiency and crossover (CO) distribution are both influenced by local and global differences in chromatin structure, yet the interplay between chromatin structure, genome integrity, and meiotic fidelity is still poorly understood. We have used the *xnd-1* mutant of *Caenorhabditis elegans* to explore the relationship between genome integrity and crossover formation. Known for its role in ensuring X chromosome CO formation and germ line development, we show that *xnd-1* also regulates genome stability. *xnd-1* mutants exhibited a mortal germ line, high embryonic lethality, high incidence of males, and sensitivity to ionizing radiation. We discovered that a hypomorphic allele of *mys-1* suppressed these genome instability phenotypes of *xnd-1*, but did not suppress the CO defects, suggesting it serves as a separation-of-function allele. *mys-1* encodes a histone acetyltransferase, whose homolog Tip60 acetylates H2AK5, a histone mark associated with transcriptional activation that is increased in *xnd-1* mutant germ lines, raising the possibility that thresholds of H2AK5ac may differentially influence distinct germ line repair events. We also show that *xnd-1* regulated *him-5* transcriptionally, independently of *mys-1*, and that ectopic expression of *him-5* suppressed the CO defects of *xnd-1*. Our work provides *xnd-1* as a model in which to study the link between chromatin factors, gene expression, and genome stability.

Genome stability encompasses the mechanisms that ensure the integrity of DNA amid constant insults, the most toxic of which are DNA double-strand breaks (DSBs). DSBs emanate from both endogenous sources (replication stress and nucleases) and exogenous sources [*e.g.*, ionizing radiation (IR)]. Induction of DSBs triggers the DNA damage response (DDR), a collection of systems that sense, respond to, and repair damaged DNA. Part of the DDR includes the initiation of cell cycle arrest to promote DNA repair, or the initiation of apoptosis if the damage cannot be repaired.

Despite their toxicity, the formation and repair of DSBs in the germ line is essential for the establishment of crossovers (COs) between homologous chromosomes during meiosis I. DSBs are purposefully created as the first step in meiotic CO formation by the topoisomerase II superfamily member Spo11 (Keeney *et al.* 1997; Dernburg *et al.* 1998). To maintain genome integrity, two events must occur: first, at least one DSB per chromosome pair must be repaired by interhomolog homologous recombination (HR) and resolved as a CO; second, additional DSBs must be repaired by HR with a non-CO outcome. Defects in either event promote genome instability, either from aneuploidy due to chromosome missegregation or through inappropriate DNA repair. Accordingly, numerous factors ensure the appropriate execution of meiotic HR, including those involved in the DDR (Lui and Colaiacovo 2013).

The covalent modification of the histone tails by a variety of posttranslational modifications (PTMs) confers exquisite variation in regulating DNA-dependent processes (Jenuwein and Allis 2001), including the DDR. Studies predominantly from mice and yeast have revealed that histone PTMs function at each step in the repair process. In response to exogenous DSBs, chromatin undergoes decondensation both locally and globally (Kruhlak *et al.* 2006; Dellaire *et al.* 2009). The relaxed chromatin structure facilitates the activation and recruitment of the kinase ATM, which initiates a signaling cascade leading to histone acetylation and additional chromatin remodeling. These modifications at the DSB site promote amplification of the DDR, recruit repair factors, and provide accessibility to the repair machinery (Deem *et al.* 2012). Following repair, acetylation of lysine 56 on histone H3 is required to inactivate the DDR (Chen and Tyler 2008) and to allow reassembly of nucleosomes at the repair site.

The interplay between chromatin structure, meiosis, and DNA repair remains a burgeoning area of research. In the *Caenorhabditis elegans* germ line, recent studies have linked global histone acetylation levels through *cra-1* to meiotic DSB formation (Gao *et al.* 2015). However, the significance of specific histone acetylation marks to DSB formation and repair is not well understood. The acetylation of lysine 5 on histone H2A (H2AK5ac) is emerging as a vital player in these processes. In *xnd-1* mutant germ lines, an increase in H2AK5ac was associated with decreased DSB formation, especially on the X chromosome (Wagner *et al.* 2010; Gao *et al.* 2015). H2AK5ac levels are diminished in *htp-3* mutants and, in late pachytene nuclei, the recycling of H2AK5ac was correlated with local synaptonemal complex remodeling and repair of IR-induced lesions from sister chromatids (Couteau and Zetka 2011). These phenotypes of H2AK5ac are regulated, at least in part, by the histone acetyltransferase (HAT) *mys-1*, the worm homolog of Tip60, the catalytic subunit of the NuA4 HAT complex. Tip60 complexes are known to modulate the DDR through acetylation of the conserved sensor, ATM. ATM, in addition to its role in the DDR, has emerged as a component of a regulatory DSB feedback loop for meiotic DSBs, raising the possibility that Tip60, ATM, and H2AK5ac may share roles in both DSB formation and repair. We have found that *xnd-1* provides a lens with which to explore the interplay between these factors in the *C. elegans* germ line.

Previously, we identified *xnd-1* as an autosomally-associated protein that regulates X chromosome CO formation through chromatin structure (Wagner *et al.* 2010); additionally, we recently described a role for *xnd-1* in germ line development (Mainpal *et al.* 2015). The variability in the severity of *xnd-1* mutant phenotypes, including brood size, lethality, and sterility, was reminiscent of mutator loci and “mortal germ line” genes, and suggested a broader role for *xnd-1* in maintaining genome stability. Here, we show that *xnd-1* is a regulator of genome stability in the *C. elegans* germ line. *xnd-1* mutants exhibited a mortal germ line phenotype and were sensitive to IR, consistent with a role in responding to DNA damage. Interestingly, a hypomorphic allele of the HAT *mys-1* completely rescued *xnd-1* IR sensitivity, improved fecundity, and restored meiotic DSB kinetics. This allele also reduced H2AK5ac levels, suggesting that these phenotypes may be a consequence of altered H2AK5ac levels. Although *mys-1*-dependent functions had no effect on the high incidence of male (Him) frequency of *xnd-1* mutants, we found instead that the X chromosome CO defect in *xnd-1* mutants was due to reduced expression of *him-5*, which XND-1 appears to regulate transcriptionally. This work suggests that XND-1 functions as both a modulator of transcription and chromatin structure, and identifies aspects of DSB formation and repair that are more acutely or less sensitive to perturbations in these functions.

## Materials and Methods

### Culture and strains

For all experiments, worms were cultured on NGM plates seeded with OP50 at 20° unless otherwise noted (Brenner 1974). Mutant strains used in this study were: LG I, *hus-1(op244)*, *atm-1(gk186)*, *cep-1(gk138)*, *cep-1(lg12501)*; LG III, *xnd-1(ok709)*; LG IV, *ced-3(n717)*; and LG V, *mys-1(n3681)*, *him-5(ok1896)*. Some strains were provided by the *Caenorhabditis* Genetics Center. *xnd-1(ok709)* was outcrossed multiple times for these studies due to long-term maintenance problems with the strain. Double and triple mutants were generated using standard genetic techniques and are listed in Supplemental Material, Table S1. Creation of transgenic animals is described below. N2 served as wild-type controls in this study. For strains containing either *xnd-1(ok709)* and/or an allele that must be balanced, F2 hermaphrodites were used unless otherwise noted. Due to some phenotypic differences between *xnd-1/qC1* and *xnd-1/hT2* populations, double and triple mutants were compared to isogenic balancer strains (*xnd-1* F2 from *hT2*-balanced stock is described in Table 1; *xnd-1* F2 from *qC1*-balanced stock is described in Table 2).

**Table 1:** General characteristics of strains used in this study

	Genotype	*N*	Avg. Eggs ± SEM	Avg. Brood ± SEM	% Lethal ± SEM (Normalized)	% Male ± SEM
A	N2	5	235.20 ± 12.72	231.00 ± 13.48	0.00 ± 1.04	0.10 ± 0.104
B	*xnd-1*	23	103.13 ± 10.97	40.39 ± 8.06	67.71 ± 4.82	15.49 ± 2.65*
C	*mys-1*	5	255.60 ± 21.73	226.20 ± 20.17	9.81 ± 1.24	0.00 ± 0.00
D	*xnd-1;mys-1*	32	151.53 ± 5.14**	114.03 ± 4.76**	23.57 ± 1.81**	13.00 ± 1.66
E	*hus-1*	8	230.75 ± 13.12	184.75 ± 12.46	18.63 ± 2.11	0.61 ± 0.19
F	*xnd-1;hus-1*	21	110.10 ± 12.56	32.71 ± 5.87	73.67 ± 3.83	26.17 ± 4.53*
G	*cep-1(gk138)*	10	192.30 ± 19.81	170.00 ± 19.88	10.93 ± 2.58	0.43 ± 0.34
H	*xnd-1;cep-1(gk138)*	26	102.15 ± 10.69	31.04 ± 4.85	70.77 ± 3.60	30.28 ± 3.41**
I	*cep-1(lg12501)*	8	213.38 ± 13.10	191.13 ± 12.25	8.74 ± 1.18	1.04 ± 0.33
J	*xnd-1;cep-1(lg12501)*	22	119.50 ± 10.40	32.27 ± 5.88	75.65 ± 3.63	34.69 ± 5.65**
K	*atm-1*	5	250.60 ± 10.17	239.60 ± 8.78	2.50 ± 0.63	0.08 ± 0.08
L	*xnd-1;atm-1*	21	135.86 ± 9.68*	67.67 ± 9.79*	51.88 ± 4.92*	10.23 ± 1.69
M	*ced-3*	8	257.38 ± 14.60	192.00 ± 14.30	24.47 ± 1.90	0.44 ± 0.21
N	*xnd-1;ced-3*	26	94.04 ± 5.32	19.77 ± 2.74	79.64 ± 2.25*	18.80 ± 2.48
O	*atm-1;mys-1*	18	174.83 ± 8.56	162.33 ± 8.37	5.57 ± 0.87	0.13 ± 0.07
P	*xnd-1;atm-1;mys-1*	23	119.26 ± 8.53	78.96 ± 7.39**	33.94 ± 3.13**	22.93 ± 2.27*

Data were collected and analyzed as described in *Materials and Methods*. % lethality is normalized to N2. * *P* < 0.05 *vs. xnd-1*; ** *P* < 0.01 *vs. xnd-1*. Avg., average.

**Table 2:** *eaIs15* [*Ppie-1*::*him-5*::*gfp*] rescues Him phenotype of *xnd-1* mutants

	Genotype	*N*	Avg. Eggs ± SEM	Avg. Brood ± SEM	% Lethal ± SEM	% Male ± SEM
A	*eaIs15*	6	228.17 ± 33.41	212.67 ± 30.96	4.69 ± 1.72	0.00 ± 0.00
B	*him-5*	9	253.33 ± 15.20	170.00 ± 9.58	31.25 ± 1.91	31.87 ± 0.98
C	*him-5;eaIs15*	8	247.75 ± 12.33	228.63 ± 11.55	5.88 ± 0.78*	0.30 ± 0.17*
D	*xnd-1*	26	109.69 ± 4.97	55.00 ± 3.60	49.71 ± 2.24	17.26 ± 2.05
E	*xnd-1;eaIs15*	24	87.79 ± 10.27	31.71 ± 5.15	71.14 ± 3.81	0.09 ± 0.08**

Data were collected and analyzed as described in *Materials and Methods*. % lethality is normalized to N2 (Table 1, row A). * *P* < 0.01 *vs. him-5*; ** *P* <0.01 *vs. xnd-1*. Avg., average.

### Clutch size/brood size/lethality/him frequency

L4 hermaphrodites of a given genotype were individually plated and transferred to a clean plate every 12 hr until egg-laying ceased. After transfer, the number of eggs and L1s on the plate were counted and recorded. Each plate was then scored for the number of adult hermaphrodites and males 3–4 d later. Timepoint data from each individual parent was combined to give total eggs, total adult brood, and total males. Percent hatching was calculated by dividing total adults by total eggs and multiplying by 100. Percent lethality was then calculated by subtracting this value from 100. Percent lethality is normalized to N2 to account for ∼2% error in egg counts. To calculate percent male, the total number of males was divided by the total number of adults and multiplied by 100. The data are presented as the mean ± SEM from isogenic parents. Statistical tests used were the Student’s *t*-test or Mann–Whitney *U* test, depending on whether or not the data were normally distributed based on the results of the D’Agostino–Pearson normality test.

### Sterility and mortal germ line analysis

For sterility assays, all progeny from several homozygous, hermaphrodite parents were plated individually. For strains containing either *xnd-1(ok709)* and/or another allele that must be balanced, F1 hermaphrodites (M+Z−) were selfed to generate homozygous (M−Z−) F2 progeny that were plated individually. Each plate was scored for the presence or absence of eggs and/or progeny 5 d postplating. Only plates in which the adult hermaphrodite was still present were included in the analysis. Hermaphrodites failing to lay a single egg were scored as sterile. Data from isogenic worms were combined to give the total numbers of sterile worms and hermaphrodites scored. To calculate percent sterility, the total number of sterile worms was divided by the total number of hermaphrodites and multiplied by 100.

The progeny of 10, independent F1 (M+Z−) animals were used to start 12 lines (M−Z−) that were passaged each generation by picking the first L4 animals to a new plate. If no progeny were present, the next generation was seeded by an immediate cousin (*i.e.*, progeny of one of the 12 lines from the same F1 parent). This step is critical for assaying generational sterility: since a subset of F2 *xnd-1* animals are sterile due to defects in PGC specification (Mainpal *et al.* 2015), random picking alone could lead to population loss. Population sizes were quantified every other generation by binning into size categories (sterile; 1–10 progeny; 10–20; 20–50; 50–100; and > 100). Populations were declared fully sterile when all 12 animals gave no progeny.

### Microarray

Day 1 hermaphrodites were dissected in 1 × sperm salts (50 mM PIPES pH 7.0, 25 mM KCl, 1 mM MgSO_4_, 45 mM NaCl, and 2 mM CaCl_2_) with 0.5 mM levamisole. Fifty distal gonads from both N2 and *xnd-1* were cleaved away from the maturing oocytes by cutting extruded gonads at the bend and collecting gonads in Trizol (Invitrogen) on ice. Samples were vortexed and frozen at −20° prior to RNA isolation and cDNA synthesis, which were performed as previously described (Fukushige and Krause 2012). Microarrays were performed at the Genomics Core of the National Institute of Diabetes and Digestive and Kidney Diseases using the *C. elegans* Genome Array (Affymetrix).

### Gene expression analysis

Approximately 1000 (DNA repair genes and *him-5*) or 500 (*eaIs4* and *eaIs15* transgenes) day 1 adults of a given genotype were washed thrice in 1 × M9 buffer (3 g/L KH_2_PO_4_, 6 g/L Na_2_HPO_4_, 5 g/L NaCl, and 1 mM MgSO_4_), resuspended in Trizol (Invitrogen), and vortexed for ∼60 sec before being flash frozen and stored at −80°. Once all the samples were collected, the samples were thawed on ice, sonicated, and RNA was isolated by chloroform extraction and isopropanol precipitation. Samples were treated with DNase (Sigma #AMPD1) and reverse transcribed into cDNA (Protoscript m-MuLV First Strand cDNA Synthesis kit, NEB #E6300S) according to the manufacturer’s instructions. Quantitative real-time PCRs were performed on the Applied Bio Systems 7300 Real Time PCR System using SYBR Green chemistry (SensiMix SYBR Hi-ROX kit, Bioline #QT-605) with transcript-specific primers designed using GETPrime and Primer3 (Table S2; Koressaar and Remm 2007; Gubelmann *et al.* 2011; Untergasser *et al.* 2012). The reference genes *rpl-32* and Y45F10D.4 (Hoogewijs *et al.* 2008) were used for normalization across samples and gene expression was analyzed using the ΔC_T_ method (Livak and Schmittgen 2001). Results are presented as the average of combined data from three (DNA repair genes and *him-5*) or two (transgenes) independent biological replicates, which in turn are comprised of three technical replicates each.

### IR sensitivity

L4 hermaphrodites were plated on each of four 6 cm plates at 30–100 worms/plate depending on genotype and IR dose. The following day, worms were exposed to 10, 50, or 100 Gy of IR from a ^137^Cs source (Gammacell1000 Elite, Nordion International Inc.). Twelve hours postirradiation, worms were individually plated and allowed to lay for 12 hr, at which point the number of eggs and L1s on the plate were counted. Each plate was scored for the number of adult progeny 3–4 d later. Survival was calculated as the total number of adult progeny divided by the total number of eggs/L1s relative to untreated worms ± SEM for a minimum of two independent experiments.

### Immunofluorescence

Day 1 adult worms were dissected in 1 × sperm salts with 1 mM levamisole and fixed in 0.5% triton/1% PFA for 5 min in a humid chamber. Slides were then freeze-cracked and immersed in 100% ethanol for 1 min. Following fixation, slides were washed in PBST (1 × PBS with 0.1% Tween) and incubated in primary antibody (rabbit α-H2AK5ac, Cell Signaling #2576, 1:100; guinea-pig anti-SYP-1, 1:2000, gift from Anne Villeneuve; rabbit anti-RAD-51 1:50000, Novus Biologicals; or mAb414 mouse anti-nuclear pore complex, Abcam, 1:1000) overnight at 4°. The next day, slides were washed and incubated in secondary antibody (α-rabbit Alexa 568, 1:2000 or α-guinea pig Alexa 488, 1:2000) for at least 2 hr at room temperature in the dark. Slides were washed three times, in 1 × PBST, 1 × PBST with DAPI, and 1× PBST, and then mounted in Prolong Gold with DAPI (Life Technologies) and visualized by confocal microscopy (Nikon Instruments).

Images were analyzed using Volocity 3D software (PerkinElmer). Quantification of H2AK5ac levels was performed by determining the ratio of intensity between anti-H2AK5ac (FITC channel) and anti-nuclear pore staining (Texas Red channel) in a 59 × 59 pixel box proximal to the transition zone as shown in Figure 4. Values for each germ line were averaged for three independent squares to confirm uniformity of staining. Final ratios are the average of at least eight gonads per genotype.

### Western blotting

For each genotype, a population of primarily adult worms was transferred from between 2 and 6 6 cm plates into a glass conical tube and washed thrice with 1 × M9. The remaining liquid was removed and the worm pellet was transferred to a 1.5 ml tube and flash frozen in liquid nitrogen. Pellets were thawed on ice, mixed with an equal volume of Laemmli Sample Buffer (Bio-Rad #161-0737) with 5% β-mercaptoethanol (Amresco M131), sonicated in a water bath for 2 min, heated at 95° for 10 min, then spun in a tabletop centrifuge for 5 min at maximum speed. Samples were resolved by 12% PAGE (TGX FastCast, Bio-Rad) and transferred to nitrocellulose in 20% methanol. The membrane was blocked in 5% nonfat milk/TBST (50 mM Tris-HCl pH 7.4, 150 mM NaCl, and 0.1% Tween-20) overnight at 4°. The next day, the membrane was washed in TBST and incubated in α-FLAG M2 (Sigma-Aldrich F1804, 1:5000 in 3% milk/TBST) for 2 hr at room temperature, followed by α-mouse HRP (1:50,000 in 3% milk/TBST) for 1 hr at room temperature. Products were visualized by enhanced chemiluminescence (ECL) (Invitrogen #WP20005) according to the manufacturer’s instructions. The membrane was then washed in mild stripping solution (200 mM glycine, 3.5 mM SDS, and 1% Tween-20, pH 2.2 adjusted with HCl) twice for 10 min each, followed by two washes in PBS for 10 min each and two in TBST for 5 min each, followed by blocking overnight as before. The membrane was incubated in α-E7 (tubulin, Developmental Studies Hybridoma Bank, 1:2000 in 0.2% milk/TBST) for 1.5 hr at room temperature, followed by α-mouse HRP (1:3000 in 0.2% milk/TBST) for 1 hr at room temperature. Products were visualized by ECL.

### Transgene construction

The *Ppie-1*::*him-5*::*gfp*::*pie-1* 3′ UTR transgene (*eaIs15*) was constructed by subcloning the genomic region containing the *him-5* open reading frame into pJK7 (John White lab) using 5′ *Spe*I and 3′ *Mlu*I restriction sites. The *Phim-5*::*him-5*::*gfp* transgene (*eaIs4*) was constructed by recombineering 2 × TY::GFP::3 × FLAG into the fosmid clone WRM0634bF01 at the C-terminus of the *him-5* coding sequence (Mainpal *et al.* 2015). All transgenes were integrated into the *unc-119(ed3)* strain using microparticle bombardment (Praitis *et al.* 2001).

### Data availability

The authors state that all data necessary for confirming the conclusions presented in the article are represented fully within the article.

## Results

### xnd-1 is required for maintaining genome stability

Initial studies identified *xnd-1* for its role in CO distribution and meiotic chromosome segregation. Mutations in *xnd-1* increased embryonic lethality (approximately half of the eggs failed to hatch) and increased the incidence of males (Him phenotype) compared to the wild type, evidence of X chromosome nondisjunction (Wagner *et al.* 2010). However, since we were concerned that *xnd-1* affects genome stability, we outcrossed our lab stocks over 10 times and confirmed these phenotypes by following F2 hermaphrodites throughout their reproductive lifespan, taking note of the total number of eggs laid by each animal (clutch size) and the subsequent brood (total number of resultant adult progeny), including the number of males. Despite substantial variation in the severity of each phenotype among *xnd-1* hermaphrodites, we observed an overall decrease in fecundity and fitness as measured by number of eggs laid compared to the wild type (Table 1, compare rows A and B) and hatching frequency, respectively. As expected, the number of males in *xnd-1* broods was markedly increased, albeit to a lower frequency than previously reported (Wagner *et al.* 2010), which we attribute to outcrossing. Consistent with previous reports, a fraction of *xnd-1* hermaphrodites were completely sterile and failed to lay a single egg (13.95%, *P* < 0.0001 *vs.* N2, Fisher’s exact test; Wagner *et al.* 2010; Mainpal *et al.* 2015).

**Figure 1:**
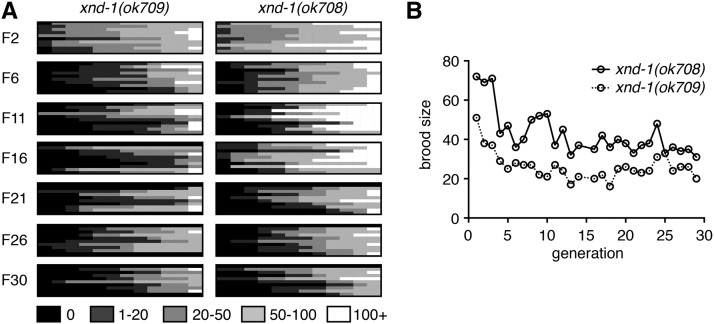
*xnd-1* exhibits a mortal germ line phenotype. (A) Heat map depicts brood sizes of 12 lines (columns) from 10 independent *xnd-1* F1s (rows) for indicated generations. Assay was performed as described in *Materials and Methods*. (B) Average brood sizes from fertile lines depicted in (A) over generations indicated. Both *ok708* and *ok709* alleles are presented and are phenotypically similar (Wagner *et al.* 2010). All other analyses are performed with *ok709*.

The variability that is observed in *xnd-1* mutants was reminiscent of mutations that cause a mortal germ line phenotype (Ahmed and Hodgkin 2000). Therefore, we set out to determine if the sterility and brood sizes associated with *xnd-1* mutations become more severe upon passaging. Fecundity was assayed in 12 lines from each of 10 independent F1 *xnd-1* animals (M+Z− from *xnd-1/hT2*) across 30 generations, or until populations were declared fully sterile (see *Materials and Methods*). As shown in Figure 1A, average brood sizes decreased and the incidence of sterility increased with progressive generations in *xnd-1* mutants. After six generations, however, the brood size appeared to level off to an average size of ∼30 progeny per worm (Figure 1B). The percentage of sterile animals, however, continued to increase over 30 generations (Figure 1A). These differences may reflect different thresholds for *xnd-1*-dependent function in egg production *vs.* offspring viability. Collectively, these results clearly indicate that *xnd-1* mutants exhibit a mortal germ line phenotype.

**Figure 2:**
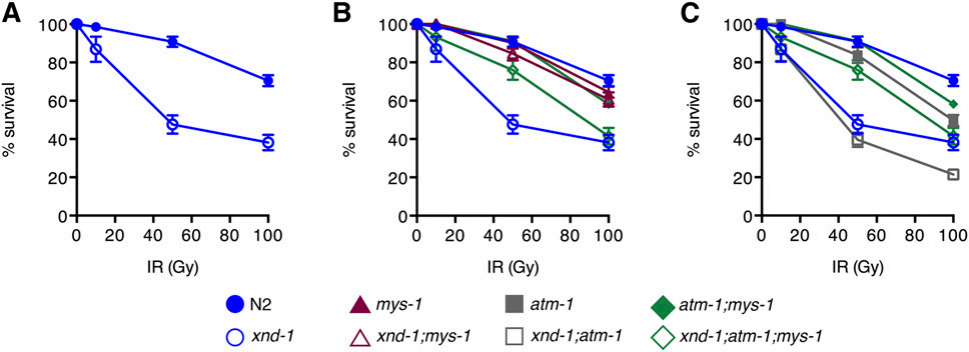
IR sensitivity of *xnd-1*, *mys-1*, and *atm-1* mutants. Progeny survival 12–24 hr postirradiation in wild-type (N2, solid blue circles), *xnd-1* (open blue circles), *mys-1(n3681)* (solid maroon triangles), *xnd-1;mys-1(n3681)* (open maroon triangles), *atm-1(gk186)* (solid gray squares), *atm-1(gk186);xnd-1* (open gray squares), *atm-1;mys-1* (solid green diamonds), and *atm-1(gk186);xnd-1;mys-1(n3681)* (open green diamonds). Data are plotted as the percent surviving progeny relative to untreated ± SEM (error bars). For easier viewing, the data are divided into N2 and *xnd-1* (A), *mys-1* and related strains in (B), and *atm-1* and related strains in (C). IR, ionizing radiation.

Close examination of brood dynamics also revealed periodic upswings and downswings in population size from single lines (Figure 1). For example, lines may have progressively decreased brood sizes over 5–10 generations, or have reached a size of fewer than 10 progeny and then increased to over 100 progeny in the next generation. Such transitions were reminiscent of our prior studies on *rfs-1*, a gene required for HR (Yanowitz 2008). Therefore, we set out to determine if *xnd-1* may have a role in responding to DNA damage. To test this, we exposed *xnd-1* F2 hermaphrodites to increasing doses of IR, which induces DSBs. The survival of progeny laid postexposure reflects the repair capacity in the hermaphrodite germ line. Survival of *xnd-1* progeny post-IR was significantly decreased compared to wild-type at both 50 and 100 Gy (*P* < 0.01 *vs.* N2 at 50 and 100 Gy, Student’s *t*-test, Figure 2A). Collectively, these results suggest that *xnd-1* is required for normal fertility, viability, and maintenance of genome stability.

**Figure 3:**
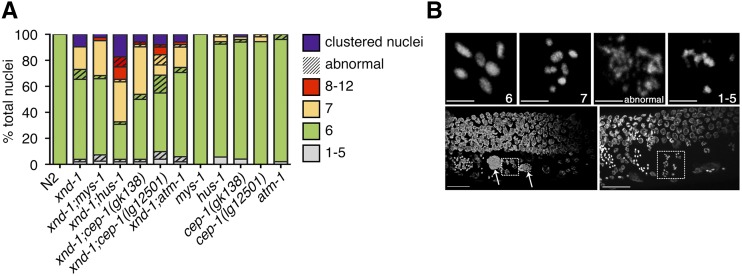
Cytological analysis and expression of select HR genes. (A) Quantification of the number of DAPI-staining bodies at diakinesis for indicated genotypes. Only the −1 oocyte was used for analysis (*n* = 40–52 nuclei). Color indicates the number of DAPI-staining bodies; hatched lines on top of a color indicate chromatin abnormalities. (B) Diakinesis phenotypes observed in oocytes of *xnd-1* mutant animals. Shown are flattened images from a confocal Z-stack of the germ line region from DAPI-stained, whole-mount, *xnd-1* mutant animals. Top row shows DNA morphologies in −1 oocytes. Scale bar, 2 µm. Bottom row shows the distal germ line on the top, proximal germ line below with sperm readily observed to the left. Large DAPI masses are marked with arrows (left); clusters of meiotic nuclei in the −2 (left) and −1 (right) oocyte positions are shown in the boxes. Normal diakinesis/diplotene nuclei can be seen in the −3 and −4 positions. Scale bar, 10 µm. DAPI, 4’,6-diamidino-2-phenylindole; HR, homologous recombination.

We hypothesized that genome instability phenotypes in *xnd-1* mutants may stem from a defect in DSB repair. First, we analyzed the quality of DSB repair by assessing DNA morphology in diakinesis-stage nuclei, as visualized by DAPI staining. In wild-type hermaphrodites, six condensed DAPI-staining bodies corresponding to six pairs of homologous chromosomes held together by chiasma are seen at diakinesis (Figure 3). Known DNA repair mutants, such as *rad-51*, exhibit decondensed chromatin and aggregates associated with defects in HR repair (Takanami *et al.* 2000; Rinaldo *et al.* 2002; Alpi *et al.* 2003). Interestingly, most *xnd-1* diakinesis nuclei observed showed either the wild-type complement of six DAPI-staining bodies or seven DAPI-staining bodies consistent with nonexchange X chromosomes (∼62 and ∼17%, respectively; Figure 3A and Wagner *et al.* 2010). In most cases, chromatin appeared to be properly condensed. However, we observed chromatin abnormalities, including aggregation, decondensation, and fragments in ∼12% of nuclei (Figure 3A). We also observed nuclei with pachytene-like morphology clustered together at the −1 oocyte position, which we called “clustered nuclei” (∼10%; Figure 3 and Wagner *et al.* 2010). The frequency of abnormal oocytes and clustered nuclei were not sufficient to account for the embryonic lethality observed in *xnd-1* mutants (Table 1, row B).

Because XND-1 protein is chromatin-associated (Wagner *et al.* 2010), we reasoned that XND-1 might alter the expression of DNA repair genes. We compared the transcriptional profile of *xnd-1* mutant *vs.* wild-type germ lines using microarrays, focusing on genes encoding factors involved in HR-mediated DSB repair (reviewed in Lemmens and Tijsterman 2011). We found three DSB repair genes (*rpa-2*, *gen-1*, and *slx-4/him-18*) that were significantly downregulated in *xnd-1* germ lines compared to wild-type (threshold of twofold, *P* < 0.05, Table S3). Consistent with their various roles in HR repair, all three factors exhibit sensitivity to DNA damaging agents (van Haaften *et al.* 2004; Saito *et al.* 2009; Bailly *et al.* 2010). We used quantitative PCRs (qPCRs) to verify the microarray data for the three DSB repair genes on our list, as well as other key genes functioning in the process such as *rad-51*, *rad-54*, and *rtel-1* as controls (Livak and Schmittgen 2001; Gubelmann *et al.* 2011). In the germ lines of Day 1 adults, all the genes we assayed were expressed similarly between *xnd-1* mutants and wild-type worms, except for *rtel-1*, whose expression was slightly reduced compared to wild-type (*P* = 0.037, Student’s *t*-test; Figure S1). As *rtel-1* mutants display greater numbers of COs (Barber *et al.* 2008), but the overall number of COs in *xnd-1* mutants is unchanged on the autosomes and reduced on the X (Wagner *et al.* 2010), it is unlikely that *rtel-1* downregulation contributes to any *xnd-1* phenotypes. Together, these results suggest that *xnd-1* mutants show phenotypes consistent with genome instability, but these do not appear to stem from misregulation of canonical DSB repair genes.

### A hypomorphic allele of mys-1 separates xnd-1 genome stability phenotypes from meiotic nondisjunction defects

The ability of *xnd-1* mutants to form a CO on the X chromosome appears to be influenced by chromatin state (Wagner *et al.* 2010). Previous studies reported increased acetylation of lysine 5 on histone H2A (H2AK5ac) in *xnd-1* mutant germ lines (Wagner *et al.* 2010; Gao *et al.* 2015). This accumulation of H2AK5ac is countered in part by RNAi against *mys-1* (Wagner *et al.* 2010), suggesting that H2AK5 is an acetylation target of MYS-1, in agreement with *in vitro* data for Tip60 (Kimura and Horikoshi 1998; Wagner *et al.* 2010). Additionally, *mys-1(RNAi)* decreases the incidence of nonexchange X chromosomes at diakinesis in *xnd-1* mutants (Wagner *et al.* 2010). To follow up on these studies and determine if *mys-1* contributes to the genome instability phenotypes of *xnd-1*, we generated *xnd-1;mys-1* double mutants. *mys-1(n4075)* is a presumptive null allele that is homozygous sterile with no diakinesis nuclei (Couteau and Zetka 2011). When combined with *xnd-1*, *n4075* was epistatic, preventing the characterization of meiotic outcomes in the double mutant. Therefore, we used *mys-1(n3681)* (hereafter referred to as *mys-1*), which encodes a missense mutation (G341R) in the acetyltransferase domain and results in viable, homozygous progeny with at least a partially functional protein (Ceol and Horvitz 2004). Compared to *xnd-1* alone, *xnd-1;mys-1* hermaphrodites had significantly increased broods due to a combination of increased egg numbers and reduced lethality, although these phenotypes were not rescued to wild-type levels (Table 1, rows B and D). The incidence of sterility in F2 homozygotes was dramatically decreased in *xnd-1;mys-1* mutants to < 1% (0.69% sterile, *P* < 0.0001 *vs. xnd-1*, Fisher’s exact test). These results indicate that *mys-1* activity negatively impacts fecundity and fitness in *xnd-1* mutants. Surprisingly, we did not observe a reduction in male frequency in *xnd-1;mys-1(n3681)* mutants as we expected, based on previous results with *mys-1(RNAi)* (Figure 3A and Table 1, 15.49% in *xnd-1 vs.* 13% in *xnd-1;mys-1*, *P* = 0.5688, Mann–Whitney) (Wagner *et al.* 2010), perhaps reflecting the weak loss-of-function nature of the *mys-1(n3681)* allele.

We next examined whether loss of *mys-1* could suppress the IR sensitivity of *xnd-1*. We noted that *mys-1(n3681)* was not sensitive to IR (Figure 2B), which is in contrast to what is observed for Tip60 function in human cell lines, where the lack of Tip60 induced IR sensitivity (Kaidi and Jackson 2013). The lack of IR sensitivity of *n3681* could be explained if the missense mutation only partially impairs acetyltransferase function or if other domains of MYS-1 are required for repair of IR-induced lesions. The lack of IR sensitivity of *mys-1* allowed us to perform epistasis with *xnd-1*. *mys-1(n3681)* completely suppressed the IR sensitivity of *xnd-1* mutant animals (Figure 2B), suggesting that *mys-1* function contributes to the genome instability phenotypes of *xnd-1*.

**Figure 4:**
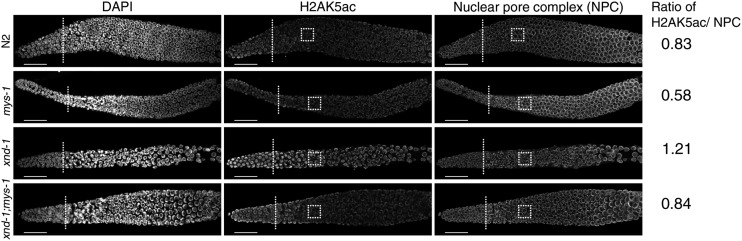
H2AK5 is an acetylation target of MYS-1. Immunofluorescence of DNA (left column), H2AK5ac (middle column), and nuclear pore complexes (right column, control) in wild-type (N2), *mys-1*, *xnd-1*, and *xnd-1;mys-1* hermaphrodite germ lines. The distal end of the germ line is oriented left, and dotted lines mark meiotic entry based on transition zone morphology. All images were taken with identical camera and laser settings and processed identically. Quantification of H2AK5ac intensity was evaluated for the boxed regions and expressed as a ratio of nuclear pore staining (see *Materials and Methods*). Scale bar, 20 μm. DAPI, 4’,6-diamidino-2-phenylindole; IR, ionizing radiation; NPC, nuclear pore complex.

As the *n3681* allele encodes a missense mutation in the acetyltransferase domain of MYS-1, we hypothesized that the improved fitness we observed in *xnd-1;mys-1* mutants could be due to decreased germ line H2AK5ac. We examined H2AK5ac in wild-type, *xnd-1*, *mys-1*, and *xnd-1;mys-1* germ lines by immunofluorescence. Consistent with previous reports, we observed elevated H2AK5ac in the mitotic zone that decreased upon entry into meiosis in wild-type germ lines, yet remained elevated upon meiotic entry in *xnd-1* germ lines (Figure 4 and Wagner *et al.* 2010; Gao *et al.* 2015). H2AK5ac was present in *mys-1* germ lines, but its levels were diminished compared to wild-type (Figure 4). Quantification of H2AK5ac levels normalized to nuclear pore content showed a 30% reduction in *mys-1* compared to wild-type. Consistent with these results, *xnd-1;mys-1* mutant germ lines also showed a ∼30% reduction in H2AK5ac intensity in the pachytene region compared to *xnd-1* alone (Figure 4), restoring H2AK5ac levels to close to wild-type. From these data, we infer that H2AK5 is an acetylation target of MYS-1 in the germ line and that *n3681* hinders MYS-1 acetyltransferase activity. The presence of H2AK5ac in mitotic nuclei and developing oocytes of *mys-1* mutants (data not shown) is consistent with prior analyses of *mys-1(RNAi)* and *mys-1(n4075)*, and suggests that additional HATs acetylate H2AK5 in these regions (Wagner *et al.* 2010; Couteau and Zetka 2011). The suppression of *xnd-1* germ line developmental defects and IR sensitivity, but not male frequency, raises the possibility that different thresholds of acetyltransferase activity exist for different MYS-1-dependent processes. Alternatively, these results could suggest that the HAT activity of MYS-1 is required to modulate *xnd-1* IR sensitivity, but that additional function(s) of MYS-1, macromolecular complex formation for example, contribute to the meiotic nondisjunction phenotypes.

### Fecundity and progeny survival in xnd-1 mutants partially depends on atm-1

Research has implicated Tip60 involvement in multiple levels of response to DNA damage, including signaling (Squatrito *et al.* 2006). Studies in mammalian cells have shown that, in response to either IR or treatment with Trichostatin A, which induces histone hyperacetylation through inhibition of class I and II histone deacetylases, Tip60 acetylates and promotes the activation of ATM kinase (Sun *et al.* 2005, 2009; Kaidi and Jackson 2013). We wondered if the reduced fitness of *xnd-1* mutants could be explained by increased DNA damage signaling triggered by inappropriate accumulation of H2AK5ac. In the germ line, mitotic proliferation arrest and increased apoptosis are responses to genotoxic stress and governed by checkpoint genes, which ultimately prevent a cell with damaged DNA from continuing through the cell cycle until either the damage is repaired or apoptosis is initiated (Gartner *et al.* 2000). In *C. elegans*, both *atm-1* and *hus-1* are required for mitotic arrest following IR, possibly through parallel pathways (Hofmann *et al.* 2002; Garcia-Muse and Boulton 2005; Stergiou *et al.* 2007). Additionally, *hus-1* is required for DNA damage-induced apoptosis through CEP-1-dependent transcriptional activation of *egl-1* (Hofmann *et al.* 2002). Both *atm-1* and *hus-1* mutants exhibit genome instability phenotypes including a mortal germ line, high incidence of males, and spontaneous mutations (Hofmann *et al.* 2002; Jones *et al.* 2012), suggesting that they have roles in responding to endogenous genotoxic stress.

We employed double mutant analysis to examine whether DNA damaged-induced checkpoint pathways were hyperactive in *xnd-1* hermaphrodite germ lines, leading to reduced fitness. Since *cep-1* is required for DNA damage-induced apoptosis, but not mitotic arrest (Derry *et al.* 2001; Schumacher *et al.* 2001), we reasoned that we could distinguish the effects of mitotic arrest and DNA damage-induced apoptosis on *xnd-1* fecundity through phenotypic differences between *atm-1*, *hus-1*, and *cep-1* double mutants. Compared to *xnd-1* alone, we observed no change in either the average clutch size of *xnd-1;hus-1* and *xnd-1;cep-1(gk138)* hermaphrodites (Table 1, rows F and H) or the proportion of sterile hermaphrodites within these populations (15.79% sterile in *xnd-1;hus-1* and 18.87% sterile in *xnd-1;cep-1(gk138)*). We observed similar results with a second allele of *cep-1*, *lg12501*, which also abrogates *egl-1* induction, although retains some function lost by the *gk138* allele (Table 1, row J, 8.33% sterile in *xnd-1;cep-1(lg12501)*, *P* = 0.09 *vs. xnd-1*, Fisher’s exact test; Schumacher *et al.* 2005; Waters *et al.* 2010). These results suggest that neither *hus-1*-mediated DNA damage-induced mitotic arrest nor *cep-1*-mediated apoptosis contribute to the fecundity of *xnd-1* hermaphrodites.

Interestingly, the frequency of male progeny in *xnd-1;hus-1* and *xnd-1;cep-1* populations was notably increased compared to *xnd-1* (Table 1, rows B, F, H, and J), which could implicate a role for *hus-1* and *cep-1* in DSB formation. If this were the case, we would expect *hus-1* and *cep-1* to enhance the *xnd-1* CO defect and increase the frequency of univalents in diakinesis oocytes. Compared to *xnd-1*, both *xnd-1;hus-1* and *xnd-1;cep-1(gk138)* exhibited an increase in seven or more DAPI-staining bodies in the −1 oocyte (Figure 3A, *P* = 0.009 for *xnd-1;hus-1 vs. xnd-1* and 0.027 for *xnd-1;cep-1(gk138) vs. xnd-1*, Z-test for proportions). Results for *xnd-1;cep-1(lg12501)* diakinesis oocytes were milder, with ∼6% of −1 oocytes exhibiting 8–12 DAPI-staining bodies, a phenotype never observed in *xnd-1* single mutants (Figure 3A). The difference in diakinesis phenotypes between *xnd-1;cep-1(gk138)* and *xnd-1;cep-1(lg12501)* are opposite those reported for these *cep-1* alleles and *him-5* (Mateo *et al.* 2016), perhaps reflecting the more complex role of *xnd-1* in DSB formation, influencing DSB timing, number, and distribution.

One explanation for the increase in males in *xnd-1;hus-1* and *xnd-1;cep-1* mutants is that DNA damage-induced apoptosis selectively eliminates nuclei that failed to receive a CO on the X chromosome. To test this, we examined physiological germ cell death, a second apoptotic pathway that culls ∼50% of germ line nuclei under normal conditions (Gumienny *et al.* 1999). Both physiological and DNA damage-induced cell death pathways rely on the core apoptotic machinery encoded by *ced-3*, *ced-4*, and *ced-9* (Gartner *et al.* 2000). Therefore, we analyzed the male frequency of *xnd-1;ced-3* double mutants to determine if apoptosis normally eliminates a subset of *xnd-1* nuclei that fail to form X chromosome COs. We observed no change in male frequency between *xnd-1* and *xnd-1;ced-3* mutants (Table 1, rows B and N), ruling out that cell death selectively eliminates *xnd-1* oocytes with nonexchange X chromosomes. Thus, these data support a role for *hus-1* and *cep-1* in DSB formation (Mateo *et al.* 2016).

In contrast to *hus-1* and *cep-1*, *atm-1* double mutants with *xnd-1* exhibited both an increase in average clutch size compared to *xnd-1* (Table 1, rows B and L, *P* = 0.03), and a significant reduction in the proportion of sterile animals (7.41% sterile, *P* = 0.0185 *vs. xnd-1*, Fisher’s exact test). *xnd-1;atm-1* mutants also exhibited increased brood size and hatching compared to *xnd-1*, although male frequency remained unchanged (Table 1, rows B and L). Together, these results implicate a function of *atm-1* as a factor contributing to fecundity and fitness in *xnd-1* mutant germ lines.

### mys-1 and atm-1 independently mediate xnd-1 phenotypes

Both *xnd-1;mys-1* and *xnd-1;atm-1* double mutants showed increased brood sizes and hatching rates compared to *xnd-1* mutants, though the improvement was more pronounced in *xnd-1;mys-1* double mutants (*P* < 0.0001 *xnd-1;mys-1 vs. xnd-1;atm-1* for both phenotypes, Mann–Whitney, Table 1, rows B, D, and L). Given the function of Tip60 in ATM kinase activation in mammalian cells (Sun *et al.* 2005, 2009; Kaidi and Jackson 2013), we hypothesized that *mys-1* and *atm-1* may be functioning in the same pathway to control genome stability in *xnd-1* germ lines. If this were the case, we would expect that *atm-1*, like *mys-1*, would suppress the IR sensitivity of *xnd-1*. Strikingly, however, IR sensitivity of *xnd-1;atm-1* mutants resembled that of *xnd-1* single mutants up to 50 Gy, suggesting that the cause of IR sensitivity in *xnd-1* mutants is independent of *atm-1* (Figure 2C). At 100 Gy IR, the sensitivity of *xnd-1;atm-1* mutants was significantly lower than that of *xnd-1* alone (*P* < 0.01, Student’s *t*-test), suggesting that *atm-1* is required for survival following IR at high doses (> 50 Gy) only. In support of this, we noted that the IR sensitivity of *atm-1* single mutants did not differ from that of wild-type worms until 100 Gy (*P* = 0.4804 at 10 Gy, *P* = 0.2715 at 50 Gy, and *P* = 0.0001 at 100 Gy).

The opposing IR sensitivities of *xnd-1;mys-1* and *xnd-1;atm-1* mutants provided an opportunity to examine an epistatic relationship between *mys-1* and *atm-1*. We generated an *xnd-1;atm-1;mys-1* triple mutant and assayed its sensitivity to IR (Figure 2, B and C). We observed that, up to 50 Gy IR, survival of *xnd-1;atm-1;mys-1* mutants resembled that of *xnd-1;mys-1* mutants, suggesting that the acetyltransferase domain of MYS-1 contributes to IR response independently of *atm-1* and supporting the conclusion that *atm-1* is not required for repair at low IR doses. By contrast, upon exposure to 100 Gy IR, survival of *xnd-1;atm-1;mys-1* mutants was intermediate between the double mutants, with survival similar to *xnd-1* mutants. These results are best explained by independent roles of *mys-1* and *atm-1* on *xnd-1* survival postexposure to IR. These results also define a threshold IR dose that necessitates *atm-1* function for survival, similar to what had previously been proposed for apoptosis induction (Stergiou *et al.* 2007).

In mammalian cells, a missense mutation in the chromodomain of Tip60 abolishes Tip60-dependent ATM activation in response to IR, yet retains housekeeping acetylation functions (Kaidi and Jackson 2013). Therefore, we wondered if *mys-1* and *atm-1* might function in additional pathways beyond those involved in survival following IR. To test this, we analyzed the clutch size, lethality, and male frequency of *xnd-1;atm-1;mys-1* triple mutants (Table 1, row P). The average clutch size of *xnd-1;atm-1;mys-1* triple mutants was significantly smaller than that of *xnd-1;mys-1* mutants but similar to *xnd-1;atm-1* mutants (Table 1, rows D, L, and P, *P* < 0.0029 *vs. xnd-1;mys-1*, *P* = 0.2308 *vs. xnd-1;atm-1*), suggesting that *atm-1* is epistatic to *mys-1* for this phenotype. However, we noticed a marked reduction in average clutch size between *atm-1;mys-1* double mutants and either single mutant (Table 1, rows C, K, and O, *P* < 0.001 *atm-1;mys-1 vs. atm-1* or *mys-1*), indicating that *atm-1* and *mys-1* function in parallel pathways to control egg production. Lethality of *xnd-1;atm-1;mys-1* mutants fell between that of *xnd-1;mys-1* and *xnd-1;atm-1* mutants, and was statistically distinct from both (*P* < 0.01 *xnd-1;atm-1;mys-1 vs. xnd-1;mys-1* or *xnd-1;atm-1*). Surprisingly, the male frequency of *xnd-1;atm-1;mys-1* triple mutants was higher than that of either double mutant or each of the single mutants (Table 1, rows B, D, L, and P, *P* < 0.001 *xnd-1;atm-1;mys-1 vs. xnd-1;mys-1* or *xnd-1;atm-1*, *P* < 0.05 *xnd-1;atm-1;mys-1 vs. xnd-1*). Consistent with the increase in male progeny, we observed an increase in the incidence of seven DAPI-staining bodies at diakinesis in *xnd-1;atm-1;mys-1* germ lines (Figure 3A, *P* = 0.19 *vs. xnd-1;mys-1*, *P* < 0.01 *vs. xnd-1;atm-1*, Z test for proportions), implicating redundant roles for *atm-1* and *mys-1* in X chromosome CO formation. Taken together, these results are most consistent with a model in which *atm-1* and *mys-1* mediate genome stability phenotypes in *xnd-1* mutants through independent mechanisms.

### xnd-1 promotes X chromosome CO formation by regulating him-5 independently of mys-1

One proposed explanation for the X chromosome CO defect in *xnd-1* mutants is that increased histone H2A lysine 5 acetylation changes the chromatin architecture such that the X chromosome is rendered inaccessible to DSBs. The previous observation that RNAi against *mys-1* increases X chromosome CO formation (Wagner *et al.* 2010) supports this hypothesis, yet is confounded here by a similar male frequency (indicative of X chromosome nondisjunction) of *xnd-1* and *xnd-1;mys-1(n3681)* broods (Table 1, rows B and D). This discord could reflect differences in *mys-1* levels and activity between *mys-1(RNAi)* and *mys-1(n3681)*; alternatively, it could reveal roles for a separate factor responsible for X chromosome CO formation.

**Figure 5:**
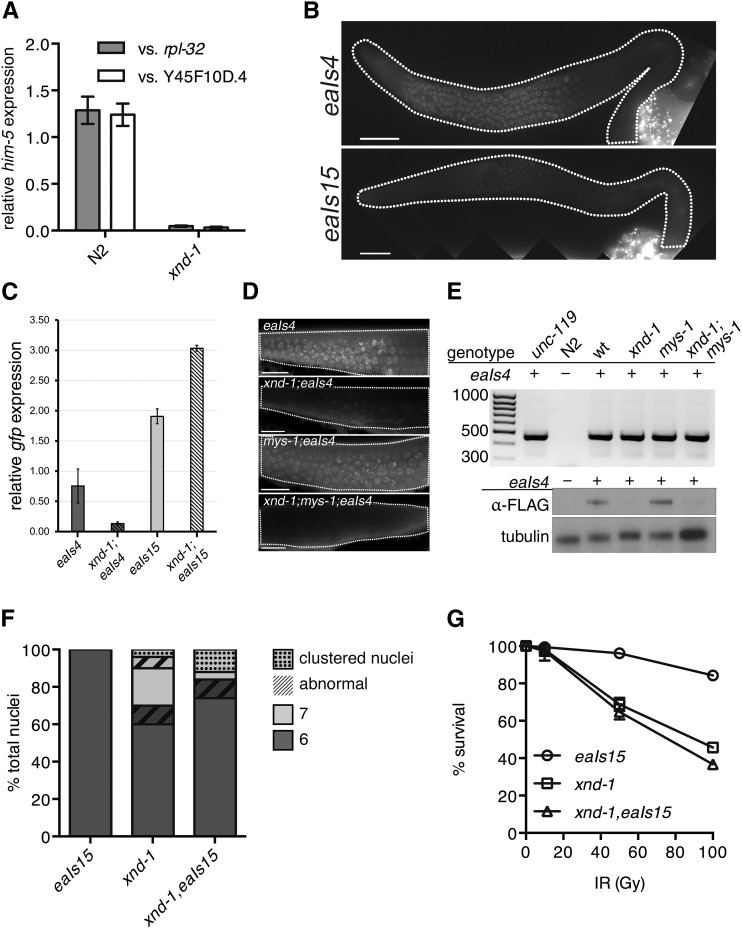
Downregulation of *him-5* is responsible for the *xnd-1* X chromosome CO defect. (A) qPCR analysis of *him-5* expression in wt (N2) and *xnd-1* hermaphrodites as compared to *rpl-2* and *y45f10d.4*. (B) Germ line expression patterns of *eaIs4* and *eaIs15* recapitulate *him-5* and *pie-1*, respectively. Images are taken with identical camera settings to directly compare expression levels. (C) qPCR analysis of *eaIs4* and *eaIs15* expression in wt and *xnd-1* hermaphrodites as compared to *y45f10d.4*. (D) Germ line expression of *eaIs4* is reduced in *xnd-1. eaIs4* is visualized live by GFP fluorescence (green) in wt, *xnd-1*, *mys-1*, and *xnd-1;mys-1* transgenic hermaphrodites. Scale bar, 10 μm. (E) *xnd-1* is required for *eaIs4* expression. Top: representative image of *eaIs4* genotyping using transgene-specific primers shows the presence of *eaIs4*. Transgenic strains (indicated by +) show PCR product at ∼450 bp, while N2 controls do not. *unc-119;eaIs4* is the founder strain, while *wt* is a wild-type control isolated from crossing *eaIs4* into *xnd-1;mys-1* mutants. Bottom: expression of *eaIs4* visualized by western blot probed with α-FLAG antibody. Tubulin serves as a loading control. (F) Quantification of the number of DAPI-staining bodies at diakinesis for indicated genotypes. Only the −1 oocyte was used for analysis (*n* = 50 nuclei/genotype). Color indicates number of DAPI-staining bodies; hatched lines on top of a color indicate chromatin abnormalities. (G) Progeny survival 12–24 hr postirradiation in *eaIs15* (wt, circles), *xnd-1* (squares), and *xnd-1;eaIs15* (triangles). The data are plotted as percent surviving progeny relative to untreated ± SEM (error bars). CO, crossover; DAPI, 4’,6-diamidino-2-phenylindole; GFP, green fluorescent protein; qPCR, quantitative polymerase chain reaction; wt, wild-type.

A previous study suggested *xnd-1* and *him-5* function in the same genetic pathway in regards to X chromosome CO formation, and that HIM-5 levels and/or localization are dependent on *xnd-1* (Meneely *et al.* 2012). To understand the nature of this regulation, we first used quantitative real-time PCR to assess the expression of *him*-5 in wild-type and *xnd-1*. We observed a dramatic reduction in *him-5* transcript levels in *xnd-1* mutants (Figure 5A, *P* < 0.001, Student’s *t*-test), raising the possibility that *him-5* expression is regulated by XND-1 transcriptionally.

To test this directly, we compared the rescuing ability of two transgenes: the first, a *him-5*::*gfp* transgene driven by its native regulatory elements (*Phim-5*::*him-5*::*gfp*::*3xFLAG*, hereafter referred to as *eaIs4*), which was integrated as a fosmid into the genome; the second, a *him-5*::*gfp* fusion expressed from the *pie-1* promoter (*Ppie-1*::*him-5*::*gfp*::*pie-1* 3′ UTR, hereafter referred to as *eaIs15*). Expression of *eaIs4* recapitulates previously described HIM-5 localization patterns (Figure 5B and Meneely *et al.* 2012). Expression of *eaIs15* recapitulates expression from *pie-1* regulatory sequences (Merritt *et al.* 2008; Christopher Merritt 2010). HIM-5::GFP proteins from *eaIs15* are diffusely localized in the nucleoplasm of mitotic zone nuclei, concentrate into puncta in the distal meiotic region, and again become diffusely nuclear near the gonad bend/diplotene (Figure S2). Diffuse nuclear staining from *eaIs15* could also be seen in embryonic blastomeres, indicative of maternal deposition of HIM-5::GFP proteins/RNAs (data not shown). qPCR from whole worms showed a twofold higher abundance of *eaIs15* transcripts in wild-type than in *eaIs4* (Figure 5C), but live imaging showed more robust, early germ line expression of *eaIs4* (Figure 5D). The disparity between RNA and protein levels in *eaIs15* is likely due to late germ line accumulation of *eaIs15* transcripts from the *pie-1* promoter (Merritt *et al.* 2008; Christopher Merritt 2010). Despite differences in protein accumulation, both transgenes conferred rescue of the Him phenotype observed in *him-5* mutants, suggesting they are fully functional (Table 2 and Table S4).

We next crossed the *him-5* transgenes into the *xnd-1* mutant background. Although PCR revealed the presence of *eaIs4* in the genome (Figure 5E), qPCR showed a marked reduction in *eaIs4* transcript level in the *xnd-1* mutant background (Figure 5C). Consistently, both western blotting of whole animals (Figure 5E) and live imaging of GFP in adult germ lines (Figure 5C) failed to detect HIM-5::GFP proteins in the *xnd-1* mutant, where these were readily observed in the wild type (Figure 5, D and E). Consistent with reduced expression, *eaIs4* failed to suppress the Him phenotype conferred by *xnd-1* (15.79% males in *xnd-1 vs.* 10.76% males in *xnd-1;eaIs4*, *P* = 0.07, Student’s *t*-test). By contrast, *eaIs15* levels were not diminished in the *xnd-1* mutants (Figure 5C) and HIM-5::GFP was robustly expressed in *xnd-1* germ lines (Figure 5D). *eaIs15* led to both a decrease in univalent X chromosomes (Figure 5F) and a reduction in the frequency of male progeny in *xnd-1* mutants (Table 2). The difference in expression and concomitant rescue capability between *him-5* expressed from its own *vs.* a heterologous promoter strongly argues that *xnd-1* directly regulates *him-5* transcriptionally. Collectively, these results indicate that a key role of *xnd-1* in CO formation is in regulating *him-5* expression.

Despite the rescue of the X chromosome CO defect, other *xnd-1* phenotypes persisted in *xnd-1,eaIs15* animals (Figure 5G and Table 2). The average clutch and brood sizes of *xnd-1,eaIs15* mutants was indistinguishable from that of *xnd-1* mutants (Table 2). We observed a small but significant decrease in hatching in *xnd-1* mutants when *eaIs15* was present, although it appears additive with the small increase in lethality observed in wild-type worms containing the transgene, suggesting that it may be due to the site of integration of the transgene. The presence of *eaIs15* had no effect on the sensitivity of *xnd-1* mutants to IR (Figure 5G). Together, these results reveal that decreased *him-5* expression accounts for only the X chromosome CO defect observed in *xnd-1* mutants. The failure of *eaIs15* to rescue other *xnd-1* phenotypes points to the involvement of multiple genes and/or pathways independent of *him-5*. Importantly, it also suggests that defects in meiotic DSB formation conferred by loss of *him-5* are not sufficient to explain the lethality observed in *xnd-1* mutants.

Having established that expression of *him-5* is controlled by *xnd-1*, we wanted to test if *mys-1* also affected *him-5* expression. Therefore, we crossed *eaIs4* into *xnd-1;mys-1* mutants and probed for *him-5* transgene expression. Similar to what we observed in *xnd-1* mutants, expression of *eaIs4* was barely detectable in *xnd-1;mys-1* mutants (Figure 5, D and E). Consistent with diminished expression, *eaIs4* was unable to rescue the Him phenotype of *xnd-1;mys-1* (11.20% males in *xnd-1;mys-1;eaIs4*). These results indicate that *him-5* expression is independent of *mys-1*-dependent changes in chromatin architecture; however, we cannot rule out the possibility that stronger reduction in *mys-1* activity would elicit changes in *him-5* expression.

### xnd-1 control of DSB timing is mys-1-dependent

**Figure 6:**
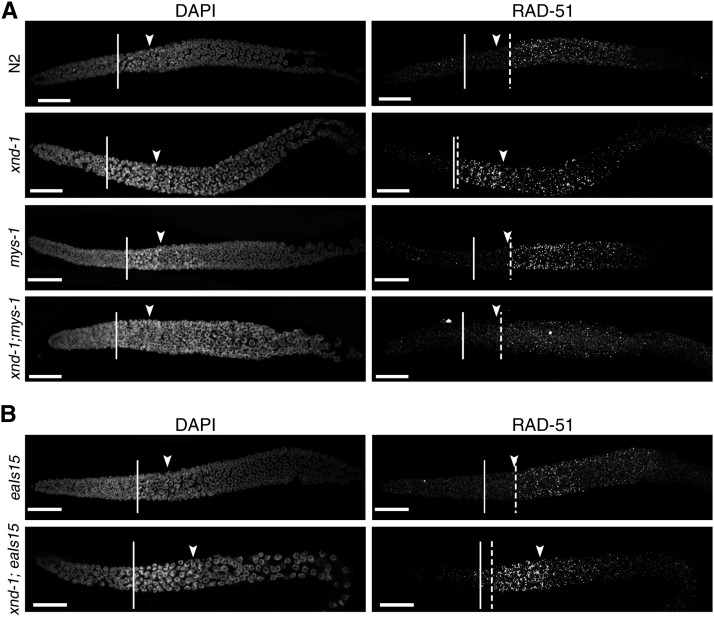
Altered dynamics of DSBs in *xnd-1* are *mys-1*-dependent. Dissected germ lines were stained with DAPI to visualize DNA and anti-RAD-51 to visualize DSB repair intermediates. The beginning of the transition zone is marked with a solid line, the end with an arrowhead; onset of significant RAD-51 foci accumulation with a dashed line. (A) Kinetics of DSBs are altered in *xnd-1* compared to wild-type, with high levels of RAD-51 appearing in the transition zone. DSB accumulation in *mys-1* is similar to wild-type, peaking in midpachytene. my*s-1* suppresses the formation of early DSBs in *xnd-1*. (B) Ectopic expression of *him-5* cannot suppress the induction of early DSBs in *xnd-1*. RAD-51 accumulation appears normal in *eaIs15* animals (top). *xnd-1* is epistatic to *eaIs15* and the double mutant shows accumulation of RAD-51 foci in the transition zone. Scale bar, 20 μm. DAPI, 4’,6-diamidino-2-phenylindole; DSB, double-strand break.

One of the most striking features of *xnd-1* meiotic germ lines is a change in the timing of DSBs (Meneely *et al.* 2012; Gao *et al.* 2015), as monitored by accumulation of the HR strand-exchange protein RAD-51 (Mets and Meyer 2009). Unlike the wild type, in which a few RAD-51 foci can be seen in transition zone nuclei but in which significant RAD-51 accumulation is not observed until midpachytene (Figure 6A), in *xnd-1*, RAD-51 accumulates in transition zone nuclei reaching peak levels in very early pachytene (Figure 6A and Meneely *et al.* 2012). Prior studies showed that this pattern of RAD-51 accumulation was independent of global histone acetylation (Gao *et al.* 2015), but its relationship to specific changes in chromatin structure were not explored. We hypothesized that the change in DSB dynamics could be a reflection of the underlying chromatin structure in *xnd-1* mutants and would therefore be suppressed by *mys-1* mutation but not by ectopic *him-5* expression (*eaIs15*), both of which alone displayed the wild-type pattern of RAD-51 accumulation (Figure 6, A and B). In support of this hypothesis, we observed that the premature accumulation of RAD-51 was suppressed in *xnd-1;mys-1* double mutants (Figure 6A) but not *xnd-1;eaIs15* (Figure 6B). These results support the model that DSB kinetics are shaped by changes in chromatin accessibility.

## Discussion

### xnd-1 is a model of genome instability

Here, we show that *xnd-1*, previously identified for its role in X chromosome DSB formation and germ line development (Wagner *et al.* 2010; Mainpal *et al.* 2015), is also an important regulator of genome stability in the *C. elegans* germ line. Homozygous *xnd-1* hermaphrodites exhibit reduced fecundity in early generations that continues to decrease over time (Figure 1), a phenotype that is characteristic of factors involved in telomere maintenance, DNA damage sensing, and chromatin modification (Ahmed *et al.* 2001; Hofmann *et al.* 2002; Andersen and Horvitz 2007; Katz *et al.* 2009). The low broods of *xnd-1* hermaphrodites result from a combination of decreased egg production and increased lethality (and perhaps reduced sperm count/function, although this has not been tested) that are independent of autosomal nondisjunction (Table 1 and Wagner *et al.* 2010). Consistent with a role in maintaining genome stability, *xnd-1* mutants are sensitive to IR (Figure 2), suggesting that *xnd-1* meiotic nuclei are unable to either properly respond to or repair exogenous DSBs. However, several observations suggest that *xnd-1* mutants are competent for DSB repair. First, induction of exogenous DSBs by low dose IR restores X chromosome CO formation (Wagner *et al.* 2010), suggesting that the DSB repair machinery is functional. Second, the majority of diakinesis oocytes exhibit well-condensed chromosomes (Figure 3A), whereas repair mutants often fail to form distinct bivalents and show massive chromosome fusions and/or DNA fragments. Third, expression of HR genes appears unaltered (Figure S1 and Table S3). Together with the observations that a hypomorphic allele of *mys-1* completely rescued *xnd-1* IR sensitivity (Figure 2B and Table 1), these results argue that the genome instability phenotypes in *xnd-1* cannot be explained by direct regulation of the HR machinery.

How does *xnd-1* deficiency lead to genomic instability, if not through regulation of HR? Our data suggest that this may be through the alteration of germline chromatin.

A missense mutation that alters the acetyltransferase domain of MYS-1 significantly improved *xnd-1* genome instability phenotypes. Our previous observation that *mys-1(RNAi)* reduced both germ line H2AK5ac and restored X chromosome CO formation in *xnd-1* mutants suggested that *mys-1* may exert its function through H2AK5ac (Wagner *et al.* 2010). The improved fecundity, fitness, and survival following IR in *xnd-1;mys-1(n3681)* mutants correlated with a reduction in H2AK5ac in meiotic nuclei, suggesting that the *n3681* allele disrupts MYS-1 acetyltransferase activity and that this activity is required for the genome instability phenotypes of *xnd-1*.

**Figure 7:**
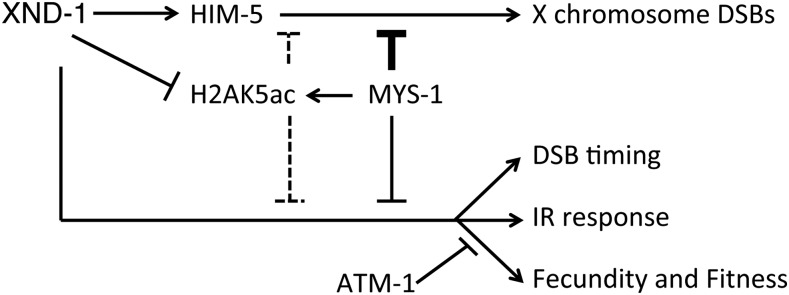
Model of genetic mediators of *xnd-1* phenotypes. In addition to defects in X chromosome DSB formation (Wagner *et al.* 2010), *xnd-1* mutants exhibit both changes in the timing of DSBs and genome instability phenotypes, including reduced fecundity and fitness and IR sensitivity. A partial loss-of-function allele of *mys-1* improves these latter phenotypes but not the X chromosome DSB defect, in part through modulation of histone H2AK5 acetylation. Loss of *atm-1* improves *xnd-1* fecundity and fitness, and does not affect survival following IR until high doses, where it appears to be generally required. Our data suggest that *mys-1* and *atm-1* function in parallel pathways. The relationship between *xnd-1*, *mys-1*, and *atm-1* is unknown. The reduced X chromosome CO formation in *xnd-1* mutants is due to downregulation of *him-5*, which *xnd-1* regulates transcriptionally. CO crossover; DSB, double-strand break; IR, ionizing radiation.

We envision several possible models to explain the relationship between H2AK5ac, *mys-1*, and *xnd-1* phenotypes (Figure 7). In the first model, XND-1 may negatively regulate *mys-1* expression. In the absence of *xnd-1*, MYS-1 levels would be upregulated leading to IR sensitivity, altered DSB kinetics, and defects in DSB formation on the X chromosome (as suggested by Wagner *et al.* 2010). The hypomorphic *mys-1* allele would reduce the increased germ line H2AK5ac levels to restore levels to near wild-type, sufficient to rescue all but the X chromosome CO defect. Regulation of *cra-1* by *xnd-1* could contribute to increased H2AK5ac levels (Lui and Colaiacovo 2013; Gao *et al.* 2015) in a *mys-1*-dependent fashion. *cra-1* promotes global histone acetylation by antagonizing acetyl-CoA hydrolase ACER-1, leading to an increased pool of acetyl-CoA which MYS-1 (and other HATs) may then utilize to increase accumulation of H2AK5ac and other histone PTMs (Gao *et al.* 2015). This model predicts that different thresholds of *mys-1* activity are required to potentiate different aspects of germ line function. Our second model proposes that *xnd-1* regulates H2AK5ac levels independently of *mys-1*, perhaps through activation of a histone deacetylase. In the absence of *xnd-1*, deacetylase activity would be impaired and H2AK5ac would accumulate. Reduction of *mys-1* function would counteract this accumulation by limiting the initial pool of H2AK5ac. In this model, different thresholds of H2AK5ac, or perhaps a balance of H2AK5ac and other acetylated histone marks, would potentiate different XND-1-dependent processes.

Finally, *mys-1* may contribute to *xnd-1* phenotypes through a mechanism apart from H2AK5 acetylation. An analysis of genetic interaction networks identified *mys-1* as a “hub” gene, defined as a gene whose loss enhanced the phenotypic consequences of mutations in unrelated genes and functional pathways (Lehner *et al.* 2006). It is also possible that the difference in restoration of X chromosome CO formation in *xnd-1;mys-1(RNAi)* mutants but not in *xnd-1;mys-1(n3681)* mutants reflects the function of different MYS-1 complexes, only one of which functions as a HAT. Further studies are needed to elucidate the MYS-1 complexes and the mechanism by which *mys-1* contributes to *xnd-1* phenotypes.

### Relationship of XND-1 and chromatin modifications to DNA repair

In late pachytene nuclei exposed to IR, H2AK5ac removal precedes localized desynapsis and correlates with intersister (IS) DSB repair, suggesting that turnover of this chromatin marks is necessary for the DDR (Couteau and Zetka 2011). By extension, we hypothesize that the decrease in H2AK5ac that we observe upon meiotic entry, a time that coincides with the onset of programmed DSB formation and HR repair, facilitates IS-HR for the excess DSBs that are made in meiotic nuclei. The elevated H2AK5ac levels in *xnd-1* mutants could impede repair by preventing access to the sister chromatid and/or by interfering with checkpoint signaling. Recent data in yeast has suggested that IS repair precedes interhomolog repair (Joshi *et al.* 2015). Although it is unknown if this temporal distinction holds true for *C. elegans*, it is tempting to speculate that the increased RAD-51 accumulation at the onset of leptotene/zygotene in *xnd-1* reflects the accumulation of DSBs destined for early IS repair events. The altered RAD-51 kinetics in *xnd-1;mys-1* double mutants further supports such a model.

The checkpoint kinase ATM/*atm-1* is also thought to function in IS repair (White *et al.* 2010; Couteau and Zetka 2011). Therefore, the increased lethality in response to IR may reflect redundant roles for these genes in IS repair. The improvements in fecundity and fitness that we observed in *xnd-1;atm-1* counter the expected relationship based on IR sensitivity. These apposing responses to *atm-1* knockdown support our supposition that the decreased brood sizes and embryonic lethality in *xnd-1* do not result directly from defects in DNA damage repair, but rather from a checkpoint signaling function of an altered chromatin state. A key signal for DNA repair in mammals is γH2AX. This alternative histone is acetylated on lysine 5 to promote its removal and attenuate DNA damage signaling (Ikura *et al.* 2007). *C. elegans* lacks H2AX homologs. It is tempting to consider the possibility that H2AK5ac may serve an analogous signaling function.

### Bipartate role for XND-1 in meiotic CO formation

Our results confirm that *him-5* is sufficient to ensure DSB formation on the X in *xnd-1* mutants (Figure 5). We found that *him-5* is downregulated in *xnd-1* mutants (Figure 5A), which explains why HIM-5 is undetectable in *xnd-1* germ lines (Meneely *et al.* 2012). Furthermore, ectopic expression of *him-5* under *pie-1* regulatory elements is sufficient to restore X chromosome CO formation (Figure 5F and Table 2), indicating that XND-1 regulates *him-5* transcriptionally.

The inability of the *Phim-5*::*him-5*::*gfp* transgene to suppress the Him phenotype of *xnd-1* (Table 1) correlates with reduced expression of the transgene in the *xnd-1* mutant background. The evidence for upregulation of *him-5* and downregulation of *cra-1* (Gao *et al.* 2013) in *xnd-1* mutant animals suggests a role for XND-1 as a transcription factor, likely working with additional cofactors to positively and negatively regulate target genes. Future studies to identify XND-1 binding partners and their impact on *him-5* and *cra-1* transcriptional targets will likely provide important mechanistic insight into XND-1 function in a variety of germ line processes.

Despite an essential role for *him-5* in promoting X chromosome COs, chromatin structure clearly still plays a critical role in establishing the recombination landscape. In wild-type germ lines, the X chromosomes receive fewer DSBs than autosomes (Gao *et al.* 2015), which may be attributed to the prevalence of repressive histone PTMs that facilitate transcriptional silencing (Kelly and Fire 1998; Kelly *et al.* 2002) limiting access to X chromosome DNA. Consistent with this model, mutations in the MES-2/3/6 complex that promotes H3K27 methylation on the X chromosome (Bender *et al.* 2004) desilence the X and suppress the Him phenotype of *xnd-1* (Wagner *et al.* 2010). Despite evidence linking increased global acetylation to increased X chromosome DSB formation (Gao *et al.* 2013), *xnd-1* mutants with increased H2AK5ac still receive dramatically fewer DSBs, both on the X chromosome and genome-wide. These data intimate that H2AK5ac hinders DSB formation. The function of this histone modification is largely unknown, but several reports implicate this mark in transcriptional activation (Shi *et al.* 2011; Rajagopal *et al.* 2014). One possibility is that H2AK5ac normally localizes to the active genes, which are found in preponderance in the central gene clusters on *C. elegans* autosomes (Barnes *et al.* 1995), facilitating their transcription but inhibiting COs. The increase in H2AK5ac levels in *xnd-1* could cause this modification to spread to ectopic sites throughout the genome. A more uniform distribution of H2AK5ac could neutralize its effect on CO inhibition and drive COs to the most open regions of the genome, the autosomal gene clusters. Alternatively, however, H2AK5ac may promote DSB formation at specific sites in the genome, and the increased expression levels and ectopic localization of this modified histone could direct DSBs to these new sites. The suppression of the X chromosome CO defect by *mys-1(RNAi)* and not *mys-1(n3681)* hints that small changes in H2AK5ac can dramatically alter CO patterns. Despite not affecting CO formation on the X chromosome, *mys-1(n3681)* did alter the kinetics of DSB formation (and repair), suggesting that chromatin structure differentially effects CO distribution, timing, and frequency. Understanding the relationship between different chromatin modifications and these meiotic processes is an area of further investigation.

## Supplementary Material

Supplemental Material
