# Supplementary material for: X Chromosome Crossover Formation and Genome Stability in Caenorhabditis elegans Are Independently Regulated by xnd-1
Source: G3 (Bethesda). 2016 Sep 27;6(12):3913–25. doi: 10.1534/g3.116.035725 (PMC5144962; doi:10.1534/g3.116.035725)
Supplement: Supplemental Material [file supp_6_12_3913__index.html]

X Chromosome Crossover Formation and Genome Stability in Caenorhabditis elegans Are Independently Regulated by xnd-1 — X Chromosome Crossover Formation and Genome Stability in Caenorhabditis elegans Are Independently Regulated by xnd-1 — Supplemental Material 

# X Chromosome Crossover Formation and Genome Stability in *Caenorhabditis elegans* Are Independently Regulated by *xnd-1*

## Supplemental Material for McClendon *et al.*, 2016

**Files in this Data Supplement:**

- Figure S1 - Quantitative PCR of select DNA repair genes. (.pdf, 3 MB)
- Figure S2 - *eaIs15* transgene expression. (.pdf, 22 MB)
- Table S1 - Strains generated for this study. (.pdf, 52 KB)
- Table S2 - qPCR primers. (.pdf, 89 KB)
- Table S3 - Fold change of HR gene transcripts in *xnd-1* germ lines vs. N2 from microarray analysis. (.pdf, 89 KB)
- Table S4 - *eaIs4* rescues the Him phenotype of *him-5*. (.pdf, 81 KB)
