# Supplementary material for: X Chromosome Crossover Formation and Genome Stability in Caenorhabditis elegans Are Independently Regulated by xnd-1
Source: G3 (Bethesda). 2016 Sep 27;6(12):3913–25. doi: 10.1534/g3.116.035725 (PMC5144962; doi:10.1534/g3.116.035725)
Supplement: Supplemental Material [file supp_g3.116.035725_FigureS1.pdf]

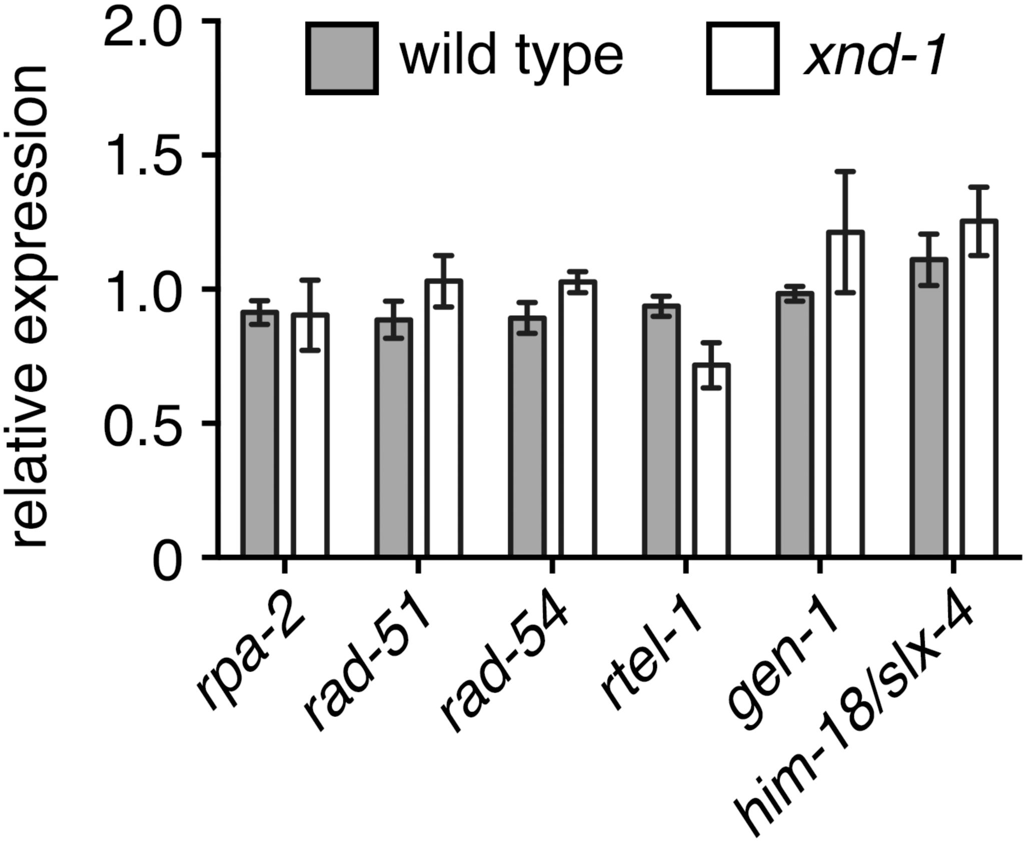

Figure S1. Quantitative PCR of select DNA repair genes.

Expression of select DSB repair genes from Supplemental Table 3 using cDNA from day 1 adult N2 or *xnd-1* hermaphrodites. Results are presented as average expression relative to reference genes from three biological replicates that in turn are comprised of three technical replicates ± SEM.
