# Supplementary material for: X Chromosome Crossover Formation and Genome Stability in Caenorhabditis elegans Are Independently Regulated by xnd-1
Source: G3 (Bethesda). 2016 Sep 27;6(12):3913–25. doi: 10.1534/g3.116.035725 (PMC5144962; doi:10.1534/g3.116.035725)
Supplement: Supplemental Material [file supp_g3.116.035725_FigureS2.pdf]

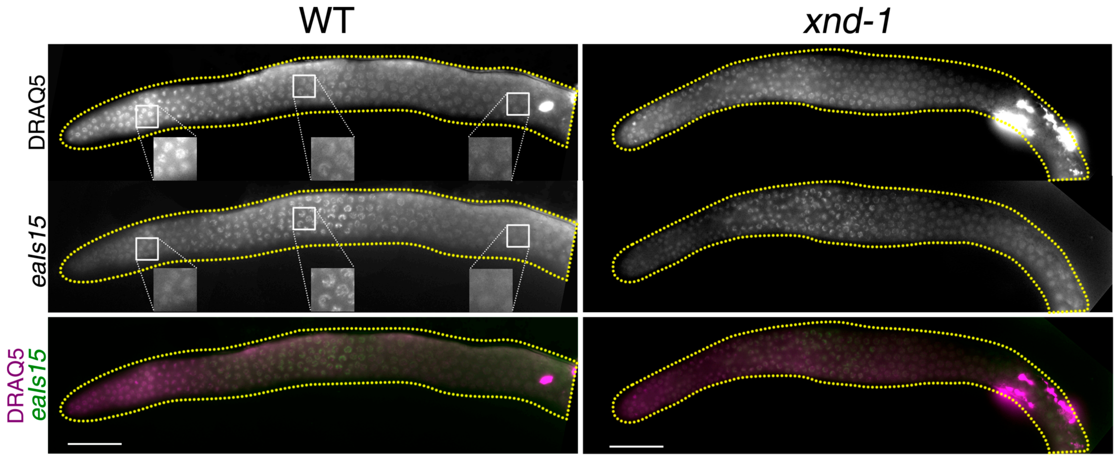

Figure S2. ***eaIs15*** **transgene expression.**

Expression of *eaIs15* (green) together with the DNA dye, DRAQ5 (magenta), reveals diffuse nuclear localization in mitotic nuclei, chromatin association in transition zone through mid-pachytene, and diffuse localization in late-pachytene (see insets). Scale bar = 20 μm.
