# Supplementary material for: X Chromosome Crossover Formation and Genome Stability in Caenorhabditis elegans Are Independently Regulated by xnd-1
Source: G3 (Bethesda). 2016 Sep 27;6(12):3913–25. doi: 10.1534/g3.116.035725 (PMC5144962; doi:10.1534/g3.116.035725)
Supplement: Supplemental Material [file supp_g3.116.035725_TableS1.pdf]

**Table S1. Strains generated for this study.**

| STRAIN | GENOTYPE | REFERENCE IN TEXT |
|---|---|---|
| QP654 | *cep-1(gk138)* I;*xnd-1(ok709)* III/hT2 [*bli-4(3937) let-?(q782)* qIs48] | *xnd-1;cep-1(gk)* |
| QP663 | *unc-119(ed3)* III*;eaIs4*[P*him-5::him-5::gfp::3xFLAG* + unc-119(+)] ? | *unc-119;eaIs4* |
| QP810 | *xnd-1(ok709)* III/qC1 [*dpy-19(e1259) glp-1(q339)* qIs26] III;*mys-1(n3681)* V | *xnd-1;mys-1* |
| QP953 | *atm-1(gk186)* I;*xnd-1(ok709)* III/hT2 [*bli-4(3937) let-?(q782)* qIs48] (I;III) | *xnd-1;atm-1* |
| QP964 | *eaIs15*[P*pie-1::him-5::gfp* + *unc-119*(+) III];*him-5(ok1896)* V | *him-5;eaIs15* |
| QP1016 | *xnd-1(ok709)* III, *eaIs15*[P*pie-1::him-5::gfp* + *unc-119*(+) III]/qC1 [*dpy-19(e1259) glp-1(q339)* qIs26] III | *xnd-1,eaIs15* |
| QP1030 | *eaIs15*[P*pie-1::him-5::gfp::pie-1* 3' UTR + *unc-119*(+)]/qC1 [*dpy-19(e1259) glp-1(q339)* qIs26] III | *eaIs15* |
| QP1042 | *xnd-1(ok709)* III/qC1 [*dpy-19(e1259) glp-1(q339)* qIs26] III | *xnd-1 (qC1)* |
| QP1089 | *xnd-1(ok709)* III/qC1 [*dpy-19(e1259) glp-1(q339)* qIs26] III;*ced-3(n717)* IV | *xnd-1;ced-3* |
| QP1173 | qC1 [*dpy-19(e1259) glp-1(q339)* qIs26] III;*eaIs4*[P*him-5::him-5::gfp::3xFLAG* + unc-119(+)] ? | *eaIs4* |
| QP1174 | *xnd-1(ok709)* III/qC1 [*dpy-19(e1259) glp-1(q339)* qIs26] III;*eaIs4*[P*him-5::him-5::gfp::3xFLAG* + unc-119(+)] ? | *xnd-1;eaIs4* |
| QP1175 | qC1 [*dpy-19(e1259) glp-1(q339)* qIs26] III;*mys-* | *mys-1;eaIs4* |

| | | |
|---|---|---|
| | *1(n3681)* V;*eaIs4*[P*him-5::him-5::gfp::3xFLAG* + unc-119(+)] ? | |
| QP1176 | *xnd-1(ok709)* III/qC1 [*dpy-19(e1259) glp-1(q339)* qIs26] III;*mys-1(n3681)* V;*eaIs4*[P*him-5::him-5::gfp::3xFLAG* + *unc-119(+)*] ? | *xnd-1;mys-1;eaIs4* |
| QP1180 | *hus-1(op244)* I;*xnd-1(ok709)* III/hT2 [*bli-4(3937) let-?(q782)* qIs48] (I;III) | *xnd-1;hus-1* |
| QP1181 | *cep-1(lg12501)* I;*xnd-1(ok709)* III/hT2 [*bli-4(3937) let-?(q782)* qIs48] | *xnd-1;cep-1(lg)* |
| QP1182 | *atm-1(gk186)* I/hT2 [*bli-4(3937) let-?(q782)* qIs48] (I;III);*mys-1(n3681)* V | *atm-1;mys-1* |
| QP1183 | *atm-1(gk186)* I;*xnd-1(ok709)* III/hT2 [*bli-4(3937) let-?(q782)* qIs48] (I;III);*mys-1(n3681)* V | *xnd-1;atm-1;mys-1* |
