# Supplementary material for: X Chromosome Crossover Formation and Genome Stability in Caenorhabditis elegans Are Independently Regulated by xnd-1
Source: G3 (Bethesda). 2016 Sep 27;6(12):3913–25. doi: 10.1534/g3.116.035725 (PMC5144962; doi:10.1534/g3.116.035725)
Supplement: Supplemental Material [file supp_g3.116.035725_TableS2.pdf]

Table S2. qPCR primers

PRIMER	GENE	SEQUENCE (5'→3')
JY-TBM-166	Y45F10D.4 (F)	TTCACTGTTCAATGCTCGC
JY-TBM-167	Y45F10D.4 (R)	CTTAGGCCTTCTTAGTCTGCT
JY-TBM-168	*rpl-32* (F)	GGATTTGGACATGCTCCTC
JY-TBM-169	*rpl-32* (R)	GATTCCCTTGCGGCTCTT
JY-TBM-207	*him-5* (F)	CTTTCTATGCAAAGCTCCGG
JY-TBM-208	*him-5* (R)	TCGTCATTGGAGTCGACAG
JY-TBM-217	*gen-1* (F)	GGAAGCTTCGTTTACGACG
JY-TBM-218	*gen-1* (R)	TCGTAATTGCATTGTGTACGG
JY-TBM-229	*rad-54* (F)	GAAGATAAGGATCGAAAGGTGC
JY-TBM-230	*rad-54* (R)	AACACCATCTCTTTGATGCG
JY-TBM-235	*rpa-2* (F)	AGAAAGCCTGACTCGAAGG
JY-TBM-236	*rpa-2* (R)	AAAGTGCTCGATCAGATTGGA
JY-TBM-237	*rtel-1* (F)	GATTTCTCGGAGTGACACTG
JY-TBM-238	*rtel-1* (R)	TGTATTCGGTCTTCGAATTCTC
JY-TBM-241	*slx-4/him-18* (F)	TCAGCTTCCAGTACCAGTG
JY-TBM-242	*slx-4/him-18* (R)	CATTTCTTCCAAGGATACAGGT
JY-TBM-251	*rad-51* (F)	GTATCACTGAGGTTTACGGAG
JY-TBM-252	*rad-51* (R)	TCGGCAATTGACACAAGAC
141GFP-For	*gfp* (F)	CAACCATTACCTGTCCACAC
140GFP-Rev	*gfp* (R)	CCCAGCAGCTGTTACAAACTC
