# Supplementary material for: X Chromosome Crossover Formation and Genome Stability in Caenorhabditis elegans Are Independently Regulated by xnd-1
Source: G3 (Bethesda). 2016 Sep 27;6(12):3913–25. doi: 10.1534/g3.116.035725 (PMC5144962; doi:10.1534/g3.116.035725)
Supplement: Supplemental Material [file supp_g3.116.035725_TableS3.pdf]

Table S3. Fold change of HR gene transcripts in *xnd-1* germ lines vs. N2 from microarray analysis.

GENE ID	GENE	FOLD-CHANGE VS. N2	P VALUE
C36A4.8	*brc-1*	-1.36	0.00337246
T07E3.5	*brc-2*	-1.44	0.0164627
C44B9.5	*com-1*	-1.31	0.0253062
F43G6.1	*dna-2*	-1.20	0.23002
F45G2.3	*exo-1*	-1.66	0.172709
T12A2.8	*gen-1*	-2.61	0.0268457
Y55B1AL.3	*helq-1*	-1.43	0.0793733
T04A11.6	*him-6*	1.06	0.832171
ZC302.1	*mre-11*	1.03	0.836952
C43E11.2	*mus-81*	-1.40	0.00451027
T04H1.4	*rad-50*	-1.35	0.0134553
Y43C5A.6	*rad-51*	-1.10	0.428344
W06D4.6	*rad-54*	-1.89	0.00290133
C30A5.2	*rfs-1*	-1.41	0.00909496
F18A1.5	*rpa-1*	-1.54	0.0762194
M04F3.1	*rpa-2*	-2.21	0.016903
F59A3.5	*rpa-3*	-1.29	0.123258
F25H2.13	*rtel-1*	-1.90	0.00602302
F56A3.2	*slx-1*	-1.88	0.00210819
T04A8.15	*slx-4/him-18*	-2.65	0.000529513
Y56A3A.27	*top-3*	1.55	0.150057
C47D12.8	*xpf-1*	-1.79	0.147465
