# Supplementary material for: X Chromosome Crossover Formation and Genome Stability in Caenorhabditis elegans Are Independently Regulated by xnd-1
Source: G3 (Bethesda). 2016 Sep 27;6(12):3913–25. doi: 10.1534/g3.116.035725 (PMC5144962; doi:10.1534/g3.116.035725)
Supplement: Supplemental Material [file supp_g3.116.035725_TableS4.pdf]

Table S4. *eaIs4* rescues the Him phenotype of *him-5*.

GENOTYPE	N	# MALE	% MALE
him-5	1640	590	36
him-5;eaIs4	350	0	0
